# Ginsenoside G-Rh2 synergizes with SMI-4a in anti-melanoma activity through autophagic cell death

**DOI:** 10.1186/s13020-018-0168-y

**Published:** 2018-02-21

**Authors:** Da-lun Lv, Lei Chen, Wei Ding, Wei Zhang, He–li Wang, Shuai Wang, Wen-bei Liu

**Affiliations:** 1 Department of Burn and Plastic Surgery, First Affiliated Hospital of Wannan Medical College, Jinghu District, Wuhu, 241000 Anhui China; 2 Dermatological Department, First Affiliated Hospital of Wannan Medical College, Jinghu District, Wuhu, 241000 Anhui China

**Keywords:** SMI-4a, G-Rh_2_, Apoptosis, Autophagy, Melanoma

## Abstract

**Background:**

Melanoma is a leading cause of cancer death worldwide, and SMI-4a and G-Rh2 exert anti-tumor activity in multiple cancer. However, SMI-4a as well as a synergistic relationship between SMI-4a and G-Rh2 in anti-melanoma capacity are still unknown. Therefore, we investigated the effects of SMI-4a and combined SMI-4a with G-Rh2 on the viability, apoptosis and autophagy of melanoma, and to preliminarily explore the underlying mechanism of SMI-4a and combined SMI-4a with G-Rh2 in inhibiting tumor growth.

**Methods:**

Cell viability was examined with cell counting Kit 8 assay and colony formation assay; Apoptosis was evaluated by flow cytometry and Caspase 3/7 activity assay; Western blotting was used to test proteins related to autophagy and the AKT/mammalian target of rapamycin (mTOR) signaling pathway; Tumor xenograft model in BALB/c nude mice was performed to evaluate the effects of SMI-4a and combined SMI-4a with G-Rh2 in anti-melanoma in vivo.

**Results:**

SMI-4a, a pharmacological inhibitor of PIM-1, could decrease cell viability, induce apoptosis, and promote Caspase 3/7 activity in both A375 and G361 melanoma cells, and SMI-4a inhibited tumor growth by inducing autophagy via down-regulating AKT/mTOR axis in melanoma cells. Furthermore, G-Rh2 amplified the anti-tumor activity of SMI-4a in melanoma cells via strengthening autophagy.

**Conclusions:**

Our results suggested that SMI-4a could enhance autophagy-inducing apoptosis by inhibiting AKT/mTOR signaling pathway in melanoma cells, and G-Rh2 could enhance the effects of SMI-4a against melanoma cancer via amplifying autophagy induction. This study demonstrates that combined SMI-4a and G-Rh2 might be a novel alternative strategy for melanoma treatment.

**Electronic supplementary material:**

The online version of this article (10.1186/s13020-018-0168-y) contains supplementary material, which is available to authorized users.

## Background

Melanoma is the highly aggressive skin cancer with mounting incidence over the past 30 years, which is associated with early metastasis, late diagnosis, and tolerance to chemotherapy in advanced stages [1]. The emergence of some novel therapies against melanoma such as genetically targeted therapies (e.g., BRAF inhibitors) and immunotherapies (e.g., PD-1/PD-L1 and CTLA-4 antibodies) has undoubtedly improved melanoma treatment, but the overall prognosis of patients remains poor [2, 3]. Therefore, it is urgently required to discover novel targets and develop more effective and lasting therapeutic strategies for melanoma.

PIM-1, a member of a newly defined class of serine/threonine kinases, has been showed to be overexpressed in multiple cancers such as prostate cancer and gastric carcinoma, and possesses oncogenic functions [4]. The overexpression of PIM-1 contributes to carcinogenesis by inhibiting apoptosis and promoting cell proliferation. Therefore, inhibition of PIM-1 activity is an emerging approach for cancer therapy. SMI-4a is a selective inhibitor of PIM-1 protein and exerts anti-tumor activity in chronic myeloid leukemia cells. In addition, PIM-1 could promote melanoma cells migration and invasion [5, 6]. However, whether SMI-4a is effective in melanoma has not been investigated.

Ginsenoside Rh2 (G-Rh2), a key bioactive component from roots of ginseng, have a wide variety of biological activities, such as anti-diabetes, anti-cancer and immune stimulation [7–9]. A recent report supported that G-Rh2 could inhibit the growth of human malignant melanoma cells by inducing apoptosis [10]. Also, G-Rh2 could inhibit hepatocellular carcinoma and cancer stem-like cells by increasing autophagy [11, 12]. Autophagy is a complex process that could degrade and recycle cellular compartments for survival during stress, whereas it may lead to cell death [13–15]. Moreover, autophagy could function as a tumor suppressor to suppress tumor growth by regulating cancer cell proliferation and apoptosis [16]. However, whether G-Rh2 could synergize with the other therapies (e.g., SMI-4a) to enhance their anti-melanoma function via inducing autophagy remains unknown.

Here, we demonstrated that SMI-4a had the anti-melanoma activity, and illustrated its potential mechanisms of activity, involving decreasing viability, enhancing apoptosis, strengthening Caspase 3/7 activity. In addition, SMI-4a inhibited the AKT/mTOR signaling axis to promote the apoptosis and autophagy. We further showed that G-Rh2 could synergize with SMI-4a in anti-melanoma capacity, possibly through enhancing autophagy both in vitro and in vivo.

## Methods

The Minimum Standards of Reporting Checklist contains details of the experimental design, and statistics, and resources used in this study (Additional file 1).

### Cell lines

Two human melanoma cell lines A375 and G361 were obtained from Cell Bank of Chinese Academy of Sciences (Shanghai, China), and propagated in DMEM supplemented with 10% heat-inactivated FBS, 100 units/ml penicillin and 0.1 mg/ml streptomycin. Both the cell lines were cultured 37 °C in a humidified atmosphere of 5% CO_2_.

### Cell viability analyses

Cell viability was tested by Cell Counting Kit-8 (CCK-8) assay (Beyotime, Shanghai, China) according to the manufacturer’s instructions. Cells (6 × 10^3^ cells/well) were treated with different concentrations of SMI-4a (0.625–10 μM) (Sigma-Aldrich, Merck KGaA, Darmstadt, Germany) or combination of G-Rh2 (Weikeqi Bioscience, China) and SMI-4a in 96-well plates for 24, 48 and 72 h, respectively. After culture, cell viability was evaluated by CCK-8 assay.

### Colony formation assays

6 × 10^2^ cells per well were propagated into six-well plates and cultured with SMI-4a (0.3 and 1 μM) for 10 days, and cells were fixed with 3.7% formaldehyde after washing with cold PBS twice. Then cells were stained with 0.5% crystal violet in methanol, and the number of colony in each well was assessed.

### Flow cytometry

Apoptosis was determined using Annexin-V-FITC/PI apoptosis detection kit (KGI Biotech, Nanjing, China) according to the manufacturer’s protocol. After SMI-4a treatment for 48 h, cells were collected, re-suspended in 500 μl of binding buffer and stained with Annexin-V-FITC and PI. The signal cells were evaluated by a FACScan (FACSCalibur, BD Biosciences, California, USA). For autophagic body production test, cells were incubated with SMI-4a (0.3, 1 and 3 μM). Cells were collected after treatment (48 h) and stained with 1 μg/ml acridine orange at room temperature (RT) for 15 min. The cells were re-suspended and analyzed by flow cytometry.

### Caspase 3/7 activity assay

Cspase-3/7 activities were tested using the Caspase-Glo^®^ 3/7 Assay kit (Promega Corporation, Madison, WI, USA) according to the manufacturer’s protocol. In brief, cells were cultured in 96-well plates (5 × 10^3^ cells/well) and treated with 1 μM SMI-4a, 3 μM G-Rh2 or combination of SMI-4a and G-Rh2 (1 + 3 μM) for 48 h. The reagent were added to cells for 1 h. At last, luminescence were measured by a microplate reader at a wavelength of 499 nm.

### Western blotting

Cells were homogenized in RIPA lysis Buffer (CST, Danvers, MA, USA) on ice. The lysates were centrifuged at 14,000×*g* at 4 °C for 15 min. The concentration of protein was tested with BCA protein assay kit (EMD Millipore, Billerica, MA, USA). After mixing with 2× Laemmli buffer and boiled for 10 min, 50 μg protein was subjected to SDS polyacrylamide gel electrophoresis, and electro-transferred onto PVDF membranes. The signals were visualized by fluorography using RapidStep™ ECL Reagent (EMD Millipore) according to the manufacturer’s directions. Antibodies recognizing GAPDH, LC3-II, Atg5, Beclin-1, AKT, p-AKT, mTOR, and p-mTOR were obtained from Cell Signalling Technology (Danvers, MA, USA).

### Tumor xenograft

Female nude BALB/c mice (6-week-old) were obtained from Vital River Laboratory (Beijing, China). 3 × 10^6^ A375 cells were injected subcutaneously into fossa axillaries of nude mice. Once the average tumor volume reached up to 200 mm^3^, the mice were randomized into four groups (n = 10) by random lottery: a control group (vehicle), a SMI-4a-treated group (15 mg/kg), an G-Rh2-treated group (30 mg/kg), and SMI-4a + G-Rh2-treated group (15 + 30 mg/kg). The tumor-bearing mice were injected intratumorally with SMI-4a, G-Rh2, or combination of SMI-4a and G-Rh2 every 2 days, and the tumor size was tested every 3 days by a caliper, and tumor volume was calculated using this formula: volume = (length × width^2^) × 1/2. We measured the tumor size and weighted the tumor samples at sacrifice. All animal protocols were performed in accordance with *the Guidelines for the care and Use of Laboratory Animals* published by the National Institutes of Health (NIH).

### Statistical analysis

Data were plotted as the mean ± S.E.M. of at least triplicate independent samples. Differences between two groups or multiple groups were analyzed by the Student’s t test or one-way ANOVA (Graphpad Prism 5). The difference was considered statistically significant when *p* < 0.05.

## Results

### SMI-4a suppressed cell growth in melanoma cells

**Fig. 1:**
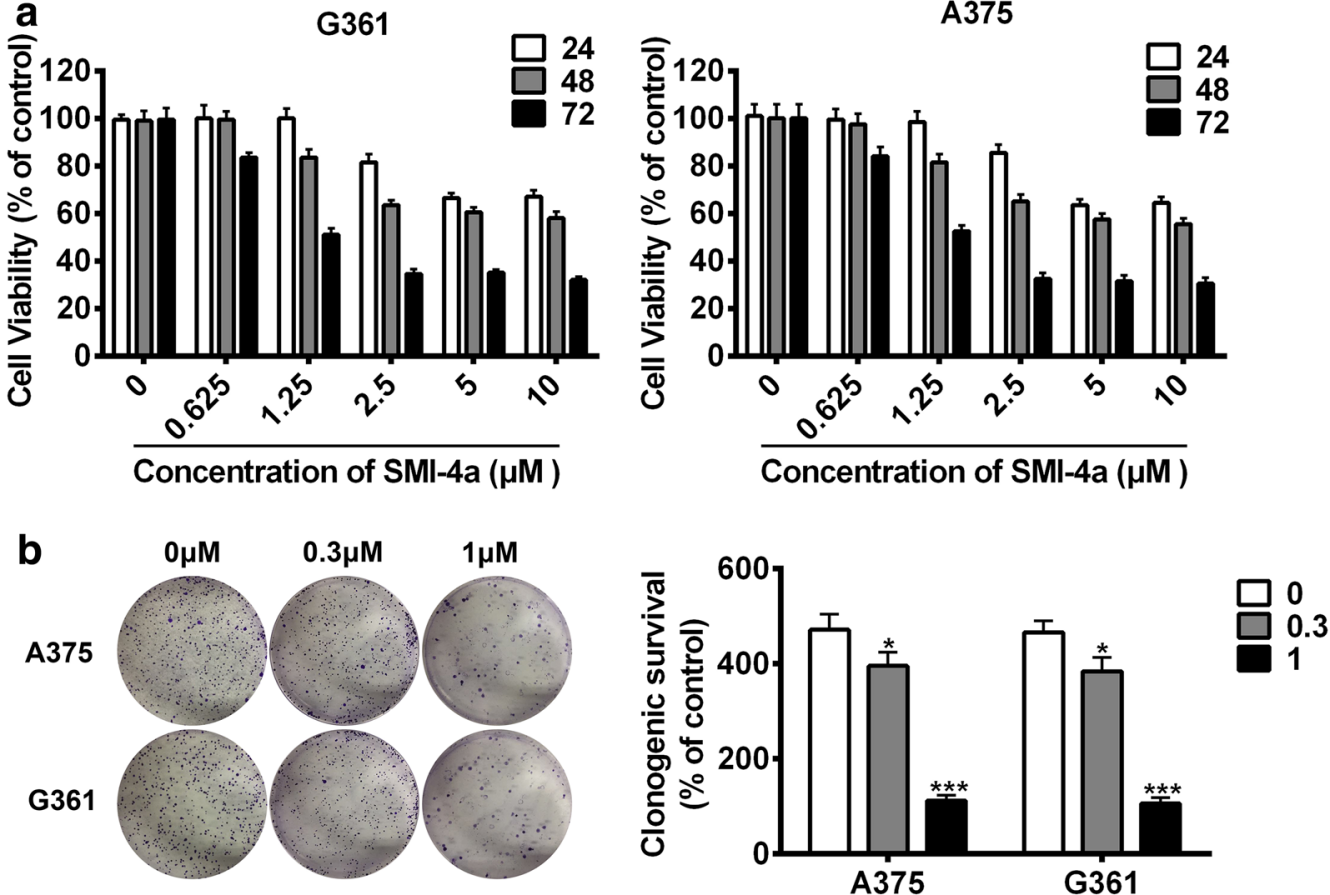
SMI-4a inhibited melanoma cell growth. **a** Cells were incubated with various dose of SMI-4a (0.625–10 μM) for 24, 48 and 72 h, respectively, and and Cells viability was tested by CCK8 assay. The results were normalized to DMSO treated cells. **b** A375 and G361 cells were treated with SMI-4a (1 and 0.3 μM) for 10 days. Colonies were fixed, stained with crystal violet and quantified. The results are expressed as the mean ± SEM. *p < 0.05 and ***p < 0.001

To investigate the effect of SMI-4a on the inhibition of human melanoma cell growth, A375 and G361 cells were cultured with various concentrations of SMI-4a for 24, 48 and 72 h, respectively. As shown in Fig. 1a, SMI-4a markedly decreased cell viability of both A375 and G361 cells in a dose- and time-dependent fashion. Similarly, SMI-4a could also remarkably inhibit cell colony formation capacities of both A375 and G361 cells in a dose-dependent fashion (*p* < 0.001, Fig. 1b). Together, these data showed that SMI-4a could retard melanoma cell growth in vitro.

### SMI-4a induced melanoma cells apoptosis

**Fig. 2:**
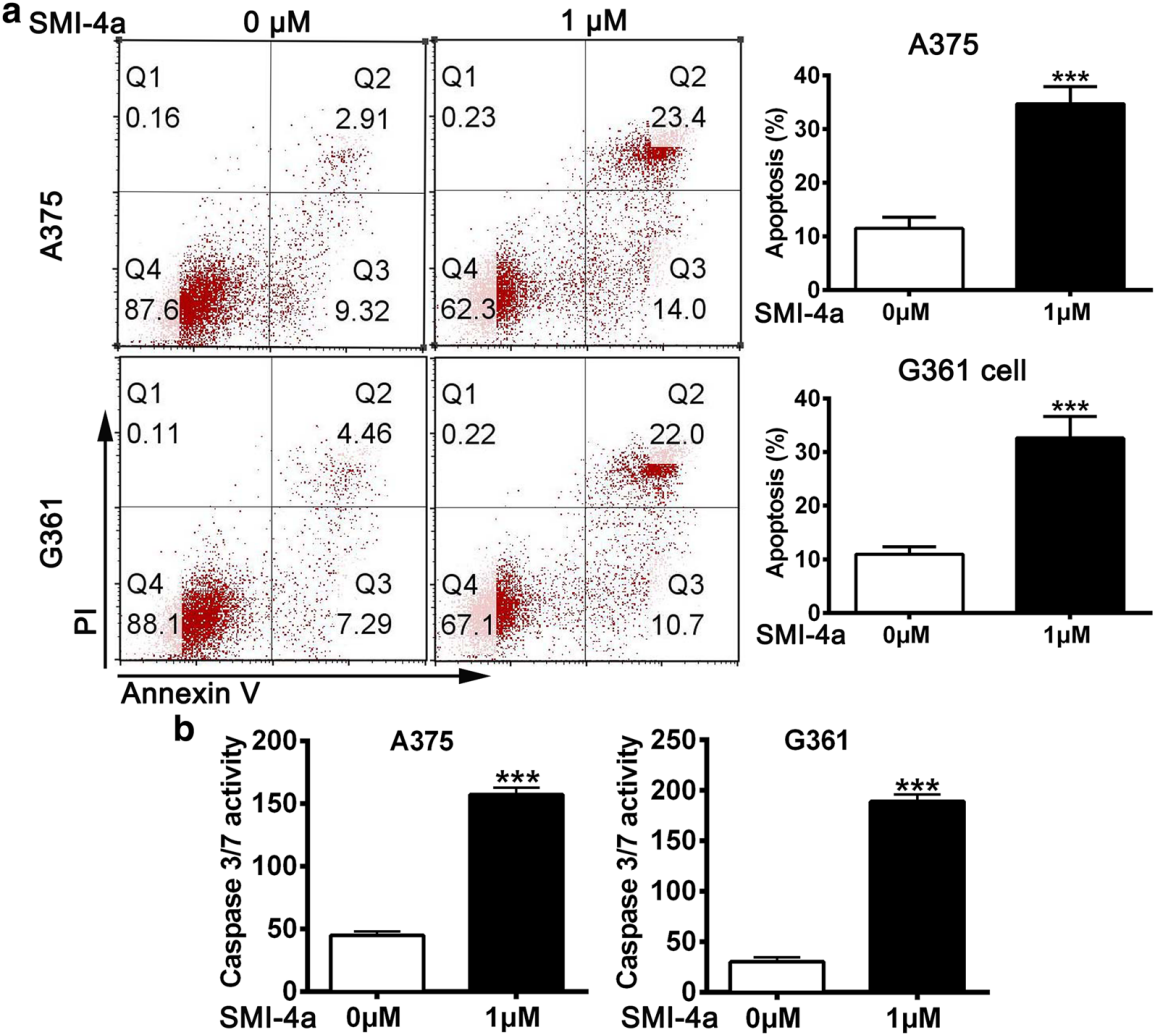
SMI-4a enhanced apoptosis in melanoma cells in vitro. **a** Flow cytometry analysis of A375 cells and G361 cells stained with Annexin V-FITC/PI after SMI-4a treatment, DMSO was used as negative control. **b** Cells were treated with SMI-4a (1 μM) for 48 h and caspase 3/7 activity was tested by the Caspase-Glo^®^ 3/7 Assay kit. The results are expressed as the mean ± SEM. *p < 0.05 and ***p < 0.001

Next, we assessed whether SMI-4a induces cell apoptosis. The flow cytometry analysis suggested that SMI-4a could dramatically promote cell apoptosis in treated A375 and G361 cells compared with the control (Fig. 2a). To further confirm the pro-apoptotic effects of SIM-4a, we also examined the activity of caspase 3/7. As shown in Fig. 2b, SMI-4a significantly enhanced the activity of caspase-3/7 in both A375 and G361 cells. These data indicate that SMI-4a could promote apoptosis of melanoma cells in vitro.

### SMI-4a trigger autophagy through Akt/mTOR signaling pathway

**Fig. 3:**
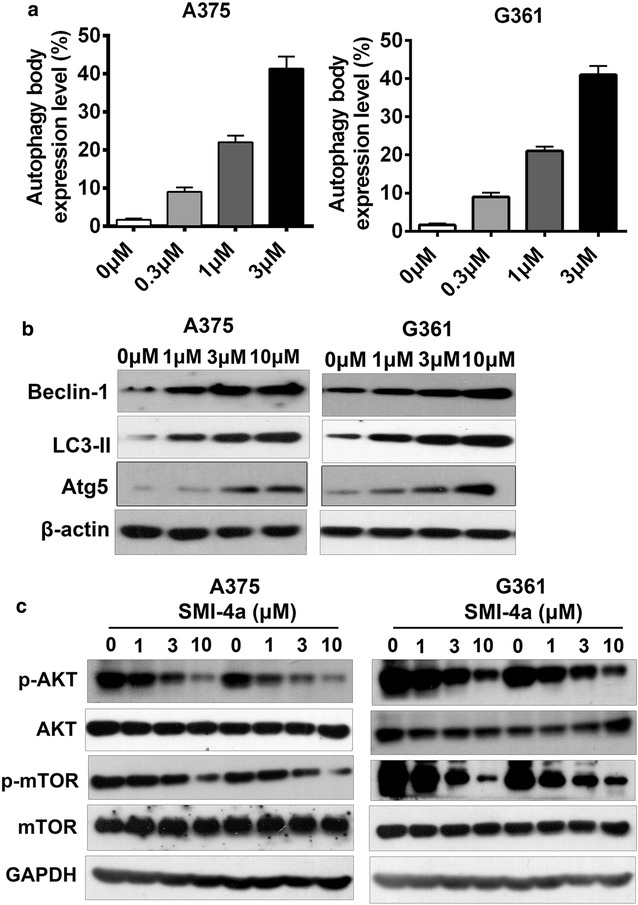
SMI-4a induced autophagy via inhibiting AKT/mTOR signaling. **a** The autophagic body formation was tested by flow cytometry analysis through staining with acridine orange, after SMI-4a treatment for 48 h. **b** Cells were treated with various doses of SMI-4a for 24 h. The protein expression of Atg5, Beclin1 and LC3-II were evaluated by a western blotting assay. **c** The levels of total AKT (AKT), phosphorylated AKT (P-AKT), Total mTOR (mTOR), phosphorylated mTOR (P-mTOR) were tested by western blot. The results are expressed as the mean ± SEM. *p < 0.05; **p < 0.01; ***p < 0.001

To examine whether SMI-4a could induce autophagy to effect cell growth, we tested autophagic body production by acridine orange (AO) staining. Intriguingly, SMI-4a markedly promoted autophagic body formation in a dose-dependent fashion compared with the control group in melanoma cells (Fig. 3a). In addition, Western blotting assay also indicated that SMI-4a enhanced the protein levels of Atg5, LC3-II and Beclin1 in a dose-dependent manner in melanoma cells (Fig. 3b), indicating that SMI-4a could induce autophagy in melanoma cells.

Next, To investigate whether the potential underlying molecular mechanisms of SMI-4a-induced autophagy is related to AKT/mTOR pathway signaling, the expressions of proteins associated with AKT/mTOR signaling were tested by western blot analysis. As shown in Fig. 3c, SMI-4a treatment decreased the phosphorylation level of Akt and mTOR without influencing the total protein amount in both A375 and G361 cells. Collectively, these data suggest that SMI-4a induces autophagy by inhibiting AKT/mTOR signaling, and consequently retards melanoma cells growth.

### G-Rh2 sensitized SMI-4a-induced cell death via enhancing autophagy in vitro

**Fig. 4:**
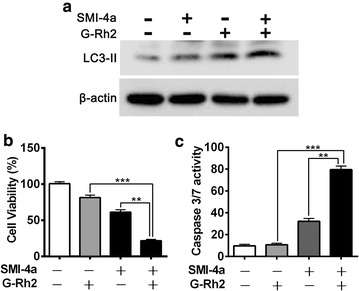
Rh2 promoted SMI-4a-induced cell death. **a** The expression of LC3-II in A375 cells were tested by Western blotting assay. **b** Cells were cultured with SMI-4a (1 μM) with G-Rh2 at 3 μM or not for 48 h, and Cells viability was tested by CCK8 assay. **c** Caspase 3/7 activity was analyzed in A375 cells treated with SMI-4a (1 μM) with G-Rh2 at 3 μM or not. The results are expressed as the mean ± SEM. *p < 0.05; **p < 0.01 and ***p < 0.001

Our results indicated that SMI-4a retarded cell growth by inducing autophagy, whereas G-Rh2 could also enhance autophagic flux. Therefore, we examined whether G-Rh2 could sensitize melanoma cells to SMI-4a-induced cell death. As shown in Fig. 4a, combination treatment with Rh2 and SMI-4a increased LC3-II protein expression, compared with Rh2 or SMI-4a treatment alone, suggesting that G-Rh2 may amplify the induction of autophagy triggered by SMI-4a. When A375 cells were pre-cultured with G-Rh2 for 1 h following by a low concentration of SMI-4a of 24 h, cells underwent significant sensitization to SMI-4a-triggered cell death (*p* < 0.001 for combination treatment versus SMI-4a alone, Fig. 4b). Furthermore, G-Rh2 also increased caspase3/7 activity by SMI-4a induction in melanoma cells (*p* < 0.001 for combination treatment versus SMI-4a alone, Fig. 4c). Together, these data suggest that Rh2 may contribute anti-tumor activity of SMI-4a in melanoma cells.

### Combination of Rh2 and SMI-4a inhibited melanoma tumor growth in vivo

**Fig. 5:**
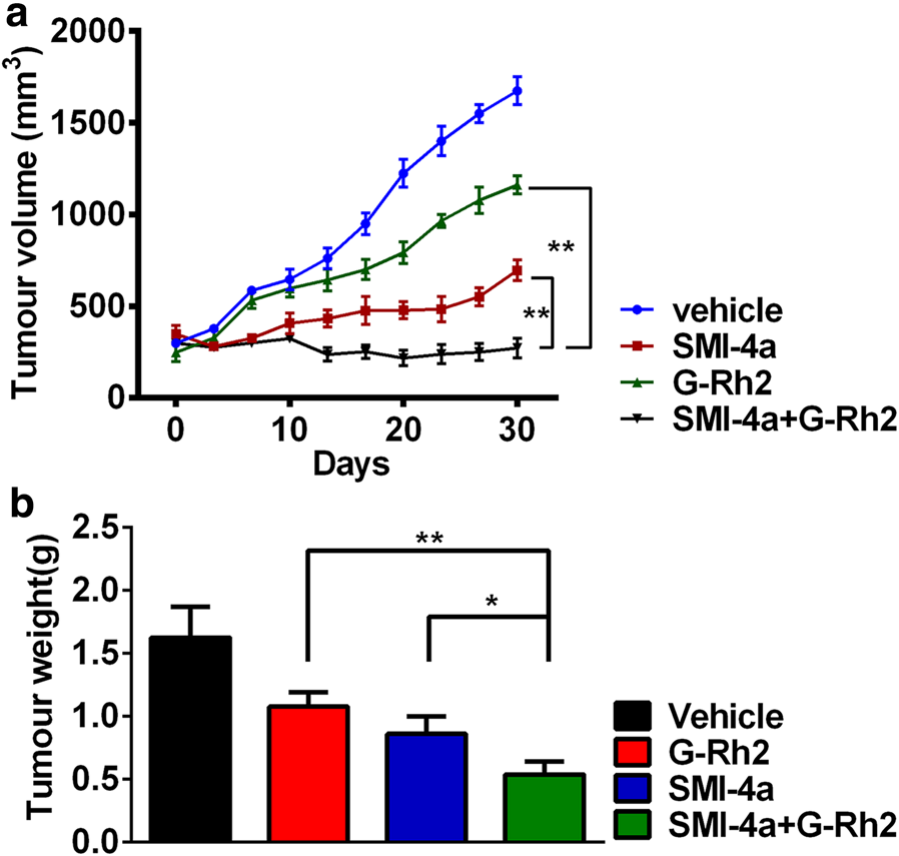
Combination treatment with Rh2 and SMI-4a inhibited melanoma growth in a mouse xenograft model. **a** and **b** A375 xenografts were treated by 0.5% MC (vehicle control), 15 mg/kg SMI-4a, 30 mg/kg G-Rh2, or combination of SMI-4a and Rh2 every 2 days for weeks. The results are expressed as the mean ± SEM. *p < 0.05 and **p < 0.01

Subsequently, to investigate the synergistic anti-tumor capacity of Rh2 and SMI-4a in vivo, we established a tumor xenograft model by subcutaneously injecting A375 cells into athymic BALB/c nude mice. Rh2 or SMI-4a treatment alone had the capacity of inhibiting tumor growth at the doses used (Fig. 5a, b). However, combination treatment with Rh2 and SMI-4a markedly suppressed tumor growth of A375 xenografts, compared with Rh2 or SMI-4a treatment alone (Fig. 5a, b). These data demonstrated that treatment of combined Rh2 with SMI-4a significantly retards tumor growth in human melanoma tumor xenografts.

## Discussion

Genetic changes of PIM-1 gene (e.g., amplifications, mutations, and deletions) are counted for about 8% of melanomas according to data of the Cancer Genome Atlas (TCGA). Silencing PIM-1 could inhibit the growth of melanoma cells [17]. In addition, SMI-4a could inhibit the growth of K562 and K562/G cells via enhancing the activity of GSK-3β [18, 19]. Similarly, in the current study we not only found that SMI-4a could dose- and time-dependently inhibit melanoma cell viability, but also dose-dependently decrease the number of colony formation in melanoma cells. Meanwhile, we also found that SMI-4a could promote cell death and increase caspase 3/7 activity in melanoma cells.

As is known to all, Akt/mTOR signaling pathway participates in multiple cellular functions, including cell survival, differentiation, and autophagy [20].The Akt/mTOR signaling pathway could negatively regulate autophagy through Akt phosphorylation, following by promoting phosphorylation and activation of mTOR [21, 22]. Notably, SMI-4a could down-regulate the phosphorylation level of Akt and mTOR. Accumulating evidence verifies that the selective inhibition of Akt/mTOR axis could suppress proliferation and invasion via enhancing autophagy in human melanoma cells [23]. Similarly, our data demonstrated that inhibition of Akt/mTOR pathway by SMI-4a markedly enhanced autophagic body formation and several key determinants of autophagy (e.g., LC3-II and Beclin-1) in a dose dependent fashion [24]. These results suggest that AKT/mTOR signaling pathway plays an important role in SMI-4a triggered autophagy in melanoma cells.

Autophagy play a complicated role in tumorigenesis, and is considered to appositive roles, pro-tumor and anti-tumor, depending on tumor cellular context. Cancer cells could utilize autophagy as a survival approach to supplement essential nutrient substances that are required for cell viability under stress. However, autophagy could not only maintain cell survival, but also may lead to cell death. Enhancing autophagy may substantially retard cancer cell growth [25–27]. Thus, SMI-4a has its inhibitory effect on melanoma cells through enhancing autophagy.

To date, many natural extracts, such as Hinokitiol and mountain tea extracts, have been shown to trigger autophagy that retard growth. Additionally, some natural extracts could synergize with some chemotherapeutic agents to eliminate cancer [28, 29]. This point was also supported in our findings showing that combined G-Rh2 with SMI-4a could markedly decrease cell viability, promote caspase 3/7 activity, and inhibit melanoma growth over their treatment alone via synergistic effects on autophagy induction, indicating that Rh2 has a synergistic effects on SMI-4a-induced anti-tumor capacity in vitro and in vivo.

## Conclusions

In short, we found that SMI-4a could decrease cell viability, promote their apoptosis in melanoma cells. We also confirmed that inhibiting Akt/mTOR signaling axis to trigger autophagy is required for SMI-4a-inducing apoptosis. Moreover, G-Rh2 could assist SMI-4a to enhance its anti-melanoma activity via strengthening autophagy-induced apoptosis in vitro and in vivo. Taken together, our results suggest that combined SMI-4a with G-Rh2 might be a novel alternative approach for melanoma treatment.

## Additional file

**Additional file 1.** The Minimum Standards of Reporting Checklist.

## Abbreviations

CCK-8: cell counting Kit 8
mTOR: mammalian target of rapamycin
P-mTOR: phosphorylated mammalian target of rapamycin
G-Rh2: ginsenoside Rh2
P-Akt: phosphorylated protein kinase B
Akt: protein kinase B
TCGA: the Cancer Genome Atlas
